# Programming effects of maternal stress on the circadian system of adult offspring

**DOI:** 10.1038/s12276-020-0398-9

**Published:** 2020-03-11

**Authors:** Seongsik Yun, Eun Jeong Lee, Han Kyoung Choe, Gi Hoon Son, Kyungjin Kim, Sooyoung Chung

**Affiliations:** 1 Department of Brain and Cognitive Sciences, Daegu Gyeongbuk Institute of Science and Technology (DGIST), Daegu, 42988 Korea; 2 Department of Biomedical Sciences, College of Medicine, Korea University, Seoul, 02841 Korea; 3 Department of Brain and Cognitive Sciences, Scranton College, Ewha Womans University, Seoul, 03760 Korea

**Keywords:** Circadian regulation, Stress and resilience

## Abstract

Maternal stress has long-lasting influences on the brain functions of offspring, and several brain regions have been proposed to mediate such programming. Although perinatal programming of crosstalk between the circadian and stress systems has been proposed, the functional consequences of prenatal stress on the circadian system and the underlying mechanisms remain largely unknown. Therefore, we investigated whether exposing pregnant mice to chronic restraint stress had prolonged effects on the suprachiasmatic nucleus (SCN), which bears the central pacemaker for mammalian circadian rhythms, of offspring. SCN explants from maternally stressed mice exhibited altered cyclic expression patterns of a luciferase reporter under control of the mouse Per1 promoter (mPer1::LUC), which manifested as a decreased amplitude and impaired stability of the rhythm. Bioluminescence imaging at the single-cell level subsequently revealed that impaired synchrony among individual cells was responsible for the impaired rhythmicity. These intrinsic defects appeared to persist during adulthood. Adult male offspring from stressed mothers showed advanced-phase behavioral rhythms with impaired stability as well as altered clock gene expression in the SCN. In addition to affecting the central rhythm, maternal stress also had prolonged influences on the circadian characteristics of the adrenal gland and liver, as determined by circulating corticosterone levels and hepatic glycogen content, and on canonical clock gene mRNA expression in those tissues. Taken together, our findings suggest that the SCN is a key target of the programming effects of maternal stress. The widespread effects of circadian disruptions caused by a misprogrammed clock may have further impacts on metabolic and mental health in later life.

## Circadian rhythms: Maternal stress can disrupt adult offspring’s biological clock

When pregnant mothers are stressed, the fetus’s circadian rhythms are reprogrammed, increasing the risk of health complications later in life. Stress during pregnancy was known to negatively affect the fetus, but how it affected circadian rhythms (day/night patterns of alertness) was poorly understood. Kyungjin Kim (Daegu Gyeongbuk Institute of Science and Technology) and Sooyoung Chung (Ewha Womans University, Seoul), both in South Korea, and co-workers stressed pregnant mice by confining them in small tubes, then measured the effects on their offspring. Pups of stressed mothers showed disturbed circadian rhythms, and the effects persisted into adulthood. Further analysis showed that the rhythms were disrupted because individual cells in the key brain region regulating circadian rhythms were poorly synchronized. These results suggest potential treatments to counteract the negative effects of prenatal stress on circadian rhythms.

## Introduction

The in utero environment can profoundly affect the life of the offspring from the fetal stage through adulthood, and these effects are known as programming effects. For example, pups from mothers who are stressed during pregnancy show low birth weights, and maternal stress has frequently been associated with the onset of various common diseases, such as metabolic, cardiovascular, and affective disorders, during adulthood, potentially owing to long-term epigenetic modifications^1,2^. Previous studies have extensively investigated the influences of maternal stress on the hypothalamus-pituitary-adrenal (HPA) neuroendocrine axis. Maternally stressed adult offspring exhibit reduced sensitivity to the negative feedback mechanisms of the HPA axis, and epigenetic alterations have been proposed to underlie these adverse outcomes^3,4^. We have also demonstrated that prenatal stress, in addition to influencing cardiometabolic and endocrine phenotypes, can have long-term consequences on cognition and emotion through impacts on several brain regions, such as the hippocampus, amygdala, ventral midbrain, and paraventricular nucleus of the hypothalamus^5–8^.

Circadian rhythm refers to biological oscillations that have a period of ~24 h and coordinate behavioral and physiological processes to facilitate adaptation to expected daily changes in the environment. This rhythm is generated by an internal timekeeping system, which is based on genetic components of a system called the circadian molecular clock^9,10^. The molecular clock is composed of a set of proteins that are required for the generation and maintenance of cell-autonomous rhythms. A key positive feedback loop of the clock is primarily controlled by the CLOCK:BMAL1 heterodimer, which activates the E-box-mediated transcription of target genes, such as *Period*s (*Pers*) and *Cryptochrome*s (*Crys*). The PER and CRY proteins translocate into the nucleus, where they inhibit CLOCK:BMAL1 heterodimer-induced transcription, including their own transcription. Nuclear receptors, such as retinoid-related orphan receptors (RORs) and REV-ERBs, are also important components of the circadian clock, establishing a stabilizing loop via their competitive actions on the ROR/REV-ERB-response element (RRE)-mediated transcription of target genes, including *Bmal1*. Importantly, the mammalian circadian system is organized in a hierarchical manner with a central pacemaker located in the suprachiasmatic nucleus (SCN) of the hypothalamus and subordinate clocks found throughout the brain and periphery^9,10^. The SCN plays a pivotal role as a master pacemaker in the regulation of circadian rhythms by receiving information on the day/night cycle, relaying temporal information to the body, and driving virtually all diurnal and circadian physiology, metabolism, and behavior. The ablation of the SCN results in the complete loss of the circadian rhythmicity of wheel-running activity and sleep-wake cycles in rodents, and the transplantation of fetal SCN tissue restores circadian locomotor rhythms with a period that is exclusively dependent on the period of the donor^11,12^. These findings strongly support the idea that the SCN is an indispensable regulator of behavioral outputs regulated by circadian rhythms.

Close links between stress and circadian systems in adulthood have been well established. For example, adrenal glucocorticoids [GCs; corticosterone (CS) in rodents and cortisol in primates] are believed to act as critical mediators of stress responses. Under stressful circumstances, the HPA axis becomes activated to respond to and cope with stress by regulating the synthesis and secretion of GCs. As the ultimate regulators of the HPA axis, GCs exert widespread actions, such as the modulation of metabolites, regulation of immune-inflammatory responses and control of higher brain functions, throughout the body^10^. In addition, GCs exhibit a rhythmic secretion pattern throughout the day regardless of stress levels, and this pattern appears to be regulated by the circadian clock^13–15^. Notably, rhythmic GC signaling contributes to the synchronization and stabilization of circadian rhythms, as revealed by locomotor activity and clock gene expression patterns in multiple peripheral tissues^14,16,17^. Therefore, altered GC signaling in a stressed mother may influence the developing circadian system of the fetus.

Indeed, maternal stress in rodents has been demonstrated to cause alterations in circadian rhythm-related phenotypes, such as advanced locomotor activity and diurnal GC rhythms, in adult offspring, suggesting the potential existence of programming effects on the circadian system caused by prenatal stress^18,19^. However, the neurobiological basis of prenatal stress-evoked alterations in circadian phenotypes remains elusive. Therefore, the present study intended to determine whether maternal stress imparts long-lasting influences on the central oscillator in the SCN and the circadian timing system of offspring.

## Materials and methods

### Animal care and handling

Wild-type and transgenic mice expressing luciferase under the mouse Per1 promoter (mPer1::LUC transgenic mice, kindly provided by Dr. Hitoshi Okamura at Kyoto University)^20^ on an ICR background were used for all experiments and were maintained in temperature-controlled (22–23 °C) quarters under a 12-h light–dark (LD) photoperiod (light on at 8:00 a.m.). Standard mouse chow and water were available ad libitum. For dark-dark (DD) conditions, mice were maintained in constant darkness for the indicated duration starting at the lights-off time. All animal procedures were approved by the Institutional Animal Care and Use Committee of Daegu Gyeongbuk Institute of Science and Technology (DGIST).

### Maternal stress procedure

**Fig. 1:**
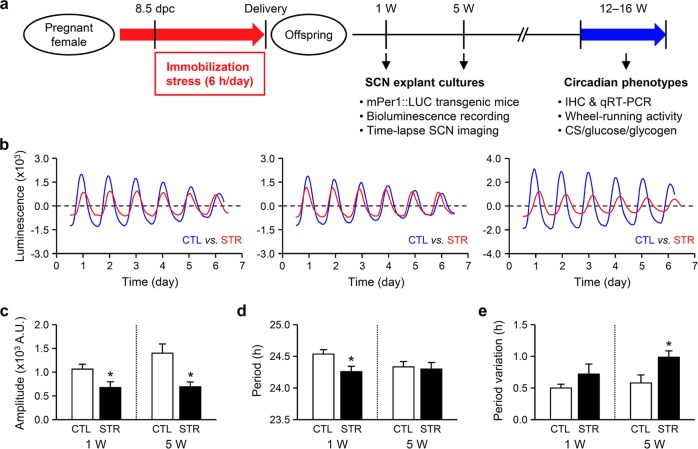
Effects of maternal stress on the circadian cycling of mPer1::LUC activity in ex vivo SCN explants. **a** A schematic summary of the experimental procedures. **b** Representative plots showing detrended traces of circadian bioluminescence signals in SCN explants prepared from 1-week-old control (CTL, blue) and maternally stressed mice (STR, red). The starting point in each plot represents 30 min before the first trough. **c**–**e** Effects of maternal stress on the amplitude (**c**), period (**d**), and periodic variation (**e**) of circadian mPer1::LUC activity in SCN cultures prepared from 1- (1 W; left) or 5-week-old (5W; right) mice. The standard deviation (SD) of the peak intervals of each SCN explant was used as an index of period variation. The data are expressed as the mean ± SE (*n* = 16 explants prepared from 1-week-old CTL mice and *n* = 10 explants prepared from 1-week-old STR mice; *n* = 8 explants prepared from 5-week-old CTL mice and *n* = 6 explants prepared from 5-week-old male STR mice; *p < 0.05 between groups by Student’s *t*-test).

The maternal stress procedure was performed as described previously^5,6^. Briefly, pregnant ICR mice were obtained by mating 6- to 7-week-old females with in-house adult males. Pregnant mice in the stress group were individually placed into a restrainer (a transparent plastic cylinder, 3 cm in diameter and 9 cm long) every day for 6 h (10:00 a.m. to 4:00 p.m.) from 8.5 days postcoitum (dpc) to 18.5 or 19.5 dpc (the day before parturition). Control pregnant mice remained undisturbed. After weaning on postnatal day (PND) 21, the pups born to stressed mothers (STR pups) were reared in an environment identical to that of control (CTL) pups. The STR and CTL groups were separately housed, but four to six mice from different litters were randomly assigned to each cage to exclude possible litter effects. Male offspring aged 12–16 weeks were used for in vivo experiments. A schematic summary of the experimental procedures is presented in Fig. 1a.

### Ex vivo SCN explant culture

Organotypic cultures were prepared from SCN slices as described in our previous studies with minor modifications^21,22^. First, mPer1::LUC transgenic mice (1 or 5 weeks of age) were sacrificed, and the brains were quickly removed. Five-week-old male offspring were used to prepare SCN explant cultures. Then, the isolated brains were chilled and placed in Gey’s balanced salt solution (GBSS) supplemented with 0.01 M HEPES and 36 mM D-glucose and bubbled with 5% CO_2_ and 95% O_2_, and then the brains were coronally sectioned into 400-µm-thick slices with a vibratome (Campden Instruments, Leicester, UK). The slices were maintained on a culture insert membrane (Millicell-CM, Millipore, Bedford, MA, USA) and placed in culture medium (50% minimum essential medium, 25% GBSS, 25% horse serum, 36 mM glucose, and 1× antibiotic-antimycotic) at 37 °C. The SCN slices were cultivated for more than 1 week before being used in experiments. All culture media and supplements were purchased from Thermo-Fisher Scientific Inc. (Waltham, MA, USA) unless otherwise specified.

### Real-time bioluminescence monitoring

The bioluminescence of the SCN slice cultures was monitored and analyzed as reported previously with minor modifications^21,22^. For quantitative measurements of bioluminescence, SCN cultures from mPer1::LUC mice were maintained in a 35-mm petri dish in 1 ml culture medium containing 0.3 mM d-luciferin (Promega, Madison, WI, USA) at 36 °C. The light emission was measured and integrated for 1 min at 10-min intervals using a dish-type wheeled luminometer (AB-2550 Kronos-Dio; ATTO, Tokyo, Japan). For single-cell recordings, time-lapse bioluminescence images were acquired at 10-min intervals using Cellgraph (ATTO). The bioluminescence data were detrended by subtracting the 24-h running average from the raw data. The peak was calculated as the highest point of the smoothed data according to CLUSTER8^23^, a statistical analysis program that identifies the significant interval values of peaks and nadirs. Period variability was examined using the standard deviation of the means for periods from each explant. The bioluminescence intensity data were normalized according to the standard deviation, and raster plots were constructed with the Hierarchical Clustering Explorer 3.5 program (available at https://www.cs.umd.edu/hcil/hce/).

### Immunohistochemistry

Mice were perfused with 4% paraformaldehyde in phosphate-buffered saline (PBS) at the indicated time of day (TOD), and the isolated brains were postfixed in the same fixative for 24 h. The brains were then cryoprotected in 30% sucrose and sectioned at 20-µm thickness on a cryostat. The SCN-containing brain sections were blocked by incubation with PBS containing 10% horse serum and 0.3% Triton X-100 for 30 min. Primary antibodies against BMAL1 (ab3350, Abcam, Cambridge, UK), PER1 (ab3443, Abcam) and PER2 (PER21-A, Alpha Diagnostic Int’l, San Antonio, TX, USA) were applied for 1 h. After washing six times with PBS, the sections were incubated with a Cy3-labeled anti-rabbit IgG antibody (Jackson ImmunoResearch Laboratory, West Grove, PA, USA) for 0.5 h. Subsequently, the sections were washed six times with PBS, mounted with 4′,6′diamidino-2-phenylindole (DAPI)-containing medium (Sigma-Aldrich, St. Louis, MO, USA) and observed under a confocal microscope (LSM510; Zeiss, Goettingen, Germany). BMAL1-, PER1- and PER2-immunoreactivity (ir) was quantitatively analyzed using the National Institutes of Health (NIH) program ImageJ (available at https://imagej.nih.gov/ij/) and then normalized against the DAPI signal intensity.

### Preparation of tissues and EDTA-plasma

Mice entrained under a 12-h LD photoperiod were maintained in constant darkness for 4 days, sacrificed by cervical dislocation at the indicated TOD, and decapitated to collect trunk blood. Tissues were isolated on ice and quickly frozen in liquid nitrogen. The SCN was bilaterally dissected from 1-mm-thick brain slices that were coronally sectioned on a brain matrix. Frozen tissues and EDTA-plasma were stored at −70 °C until use.

### RNA isolation and quantitative reverse transcription-polymerase chain reaction

RNA analyses were performed as described previously with modifications^15^. Total RNA was isolated using RNeasy Mini kits according to the manufacturer’s instructions (Qiagen GmbH, Hilden, Germany). For quantitative reverse transcription-polymerase chain reaction (qRT-PCR), 500 ng of each RNA sample was reverse-transcribed with MMLV reverse transcriptase (Promega). Then, aliquots of cDNA were subjected to qRT-PCR in the presence of SYBR Green I (Sigma-Aldrich). The gene expression levels were normalized against the mRNA expression profiles of TATA-binding protein (TBP). The primer sequences used for qRT-PCR were as follows: *Clock* up, 5′-TTG CTC CAC GGG AAT CCT T-3′; *Clock* dn, 5′-GGA GGG AAA GTG CTC TGT AG-3′; *Bmal1* up, 5′-GGC CAT CAG TTA AGG TGG AA-3′; *Bmal1* dn, 5′-GGT GGC CAG CTT TTC AAA TA-3′; *Per1* up, 5′-GTG TCG TGA TTA AAT TAG TCA G-3′; *Per1* dn, 5′-ACC ACT CAT GTC TGG GCC-3′; *Per2* up, 5′-GGA GTG CAT GGA GGA GAA AT-3′; *Per2* dn, 5′-CGA ATC TCA TTC TCG TGG TG-3′; *Rev-erbα* up, 5′-AGG GCA CAA GCA ACA TTA CC-3′; *Rev-erbα* dn, 5′-CAC AGG CGT GCA CTC CAT AG-3′; *Rev-erbβ* up, 5′-CCA TCA TGA GGA TGA ACA GG-3′; *Rev-erbβ* dn, 5′-CAA ATC GAA CAG CAT CTC GT-3′; *Tbp* up, 5′-GGG AGA ATC ATG GAC CAG AA-3; and *Tbp* dn, 5′- CCG TAA GGC ATC ATT GGA CT-3′.

### Analysis of circadian wheel-running activity

Circadian wheel-running activity was examined as described in a previous study with some modifications^14,24^. Briefly, mice were individually housed in a light-proof clean animal rack cabinet (Shin Biotech, Seoul, Korea) with a light intensity of 350–450 lux coming from the bottom of the cage during the light phase. Wheel-running activity was measured by a magnetic bar and switch placed on the side of a running wheel (12 cm in diameter and 5.4 cm in width). To measure the free-running period of wheel-running activity rhythm, entrained mice were first housed under a LD cycle for 15 days and then subjected to DD conditions. Wheel-running data were continuously recorded at 6-min intervals using the VitalView Data Acquisition System, and individual actograms were obtained using ActiView software (Starr Life Sciences, Oakmont, PA, USA). The period, amplitude, and acrophase of circadian wheel-running activity were calculated using Cosinor software (available at http://www.circadian.org). The activity onset variability was determined by averaging the differences between activity onset and best-fit regression for each day deduced by Cosinor analysis.

### Measurements of plasma CS levels, plasma glucose concentrations, and hepatic glycogen content

CS concentrations in EDTA-plasma were determined using a commercial radioimmunoassay (RIA) kit (Diagnostic Products Corporation, Los Angeles, CA, USA) as described previously^14,15^. Plasma glucose levels were measured using a FreeStyle blood glucose meter (Therasense, Uppsala, Sweden). Liver glycogen levels were evaluated based on the levels of glucose in the hydrolyzed liver as described previously^25^. Liver tissues were hydrolyzed with 2 M HCl for 4 h, and then lysates were neutralized with 2 M NaOH and 1 M Tris (pH 7.4). Glucose levels in the hepatic lysates were normalized against the DNA concentrations in each liver lysate.

### Statistical analysis

Student’s *t*-test was used for simple comparisons between groups. Two-way analysis of variance (ANOVA) followed by Bonferroni post hoc comparisons was used to statistically evaluate protein and mRNA expression levels, plasma CS profiles, hepatic glycogen content, and plasma glucose levels. Two-way repeated-measures ANOVA (RM-ANOVA) followed by Bonferroni post hoc test was employed to evaluate wheel-running activity. Correlations between period and amplitude in the bioluminescence imaging experiment were statistically evaluated by linear regression accompanied by Pearson’s correlation. A *p*-value <0.05 was considered to be statistically significant.

## Results

### Circadian mPer1::LUC expression in SCN cultures from maternally stressed mice

To test the idea that maternal stress has a long-lasting influence on the central clock of the circadian system, we examined the circadian characteristics of SCN explant cultures prepared from maternally stressed (STR) offspring. For this purpose, we prepared organotypic SCN cultures from 1- or 5-week-old mPer1::Luc transgenic mice^20^. Compared with the unstressed control (CTL) mice, which showed a robustly oscillating mPer1::LUC expression pattern, the SCN cultures prepared from neonatal STR mice showed attenuated mPer1::LUC rhythms (Fig. 1b). Accompanying analyses of circadian parameters revealed that the amplitudes of the periodic bioluminescence signals were significantly reduced in the STR SCN cultures compared with those in the CTL cultures at both ages (Fig. 1c; 1066.69 ± 102.57 A.U. for 1-week-old CTL mice vs. 679.38 ± 121.51 A.U. for 1-week-old STR mice and 1403.33 ± 193.21 A.U. for 5-week-old CTL mice vs. 696.00 ± 101.01 A.U. for 5-week-old STR mice, *p* < 0.05 between groups at the same age). SCN cultures from 1-week-old STR mice exhibited a slightly but significantly shortened period of mPer1::LUC activity compared with that of SCN cultures from age-matched CTL mice, while a similar difference was not observed in the explants prepared from 5-week-old animals (Fig. 1d; 24.54 ± 0.07 h for 1-week-old CTL mice vs. 24.26 ± 0.09 h for 1-week-old STR mice, *p* < 0.05; 24.24 ± 0.07 h for 5-week-old CTL mice vs. 24.30 ± 0.10 h for 5-week-old STR mice, *p* = 0.62). In contrast, impaired period stability was more apparent in SCN cultures prepared from older STR mice (Fig. 1e). Although the SCN cultures from 1-week-old STR mice showed a tendency toward increased period variation, the difference between groups was not significant (0.50 ± 0.06 h for 1-week-old CTL mice vs. 0.72 ± 0.16 h for 1-week-old STR mice, *p* = 0.14). The period variation observed in the cultures from 5-week-old STR mice, however, was significantly larger than that observed in cultures from age-matched CTL mice (0.58 ± 0.13 h for 5-week-old CTL mice vs. 0.99 ± 0.10 h for 5-week-old STR mice, *p* < 0.05).

**Fig. 2:**
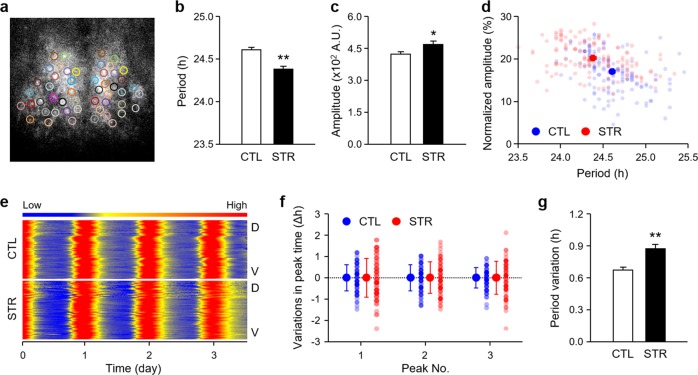
Bioluminescence imaging of circadian mPer1::LUC expression at the single-cell level in the SCN cultures. **a** The video micrograph illustrates chosen cells from an SCN culture. **b**, **c** Effects of maternal stress on the period (**b**) and amplitude (**c**) of circadian mPer1::LUC activity at the single-cell level. The data are expressed as the mean ± SE (*n* = 140 cells from two independent slices per group; **p < 0.01 between CTL and STR by Student’s *t*-test). **d** Scatter plot showing the normalized amplitudes relative to the average bioluminescence signal intensity and period of mPer1::LUC expression in individual cells from SCN explants from CTL (blue) and STR (red) mice. **e** Raster plot presentation displaying the cellular luciferase activity measured dorsal (D) to ventral (V) throughout a given SCN slice. Red corresponds with peak bioluminescence, and blue corresponds with the trough. **f** Phase differences in the peak times of each cell compared with the mean value. The x-axis represents the order of peaks. The first three peaks are shown here. The y-axis represents differences in the peak times of individual cells. Individual values are shown as dots. **g** Differences in period variations between groups are expressed as the mean ± SE (*n* = 140 cells from two slices per group; **p < 0.01 between CTL and STR, by Student’s *t*-test). The SD of peak intervals in each SCN explant was used as an index of period variation.

We then examined the circadian mPer1::LUC activity at the level of single SCN cells using time-lapse bioluminescence imaging to elucidate the cellular basis of the attenuated circadian rhythmicity in STR SCN cultures (Fig. 2a). In agreement with the results obtained from whole SCN tissues, individual SCN cells in SCN cultures prepared from 1-week-old STR pups exhibited a significantly shorter period of cyclic luciferase expression than that exhibited by SCN cells from age-matched CTL mice (Fig. 2b; 24.61 ± 0.03 h for CTL mice vs. 24.38 ± 0.03 h for STR mice, *p* < 0.01). Surprisingly, the bioluminescence signal in each cell showed even higher amplitudes in the STR SCN cells than in the CTL SCN cells, in contrast with the reduced amplitude of mPer1::LUC rhythm observed in whole STR SCN explants (Fig. 2c; 422.71 ± 13.18 A.U. for CTL explants vs. 469.15 ± 16.63 for STR explants, *p* < 0.05). A negative correlation between the period of the mPer1::LUC rhythm and the normalized amplitude relative to the average signal intensity of each cell implied that a shortened period and increased amplitude may occur concomitantly in STR SCN cells (Fig. 2d; *r* = −0.38, slope = −7.48, *p* < 0.01). This discrepancy in the amplitude appears to be related to impaired synchrony among cells. Indeed, the peak times observed for individual cells were more variable in the STR SCN cultures than in the CTL SCN cultures (Fig. 2e, f). Accordingly, period variations were significantly increased in STR SCN cells compared to CTL SCN cells (Fig. 2g; 0.67 ± 0.03 h for CTL cells vs. 0.87 ± 0.04 h for STR cells, *p* < 0.01). These findings suggest that prenatal exposure to maternal stress may cause intrinsic defects in the SCN of offspring and that these defects are linked with impaired synchrony among individual pacemaker neurons and can persist even after birth.

### Expression of core clock genes in the adult SCN of male STR mice

**Fig. 3:**
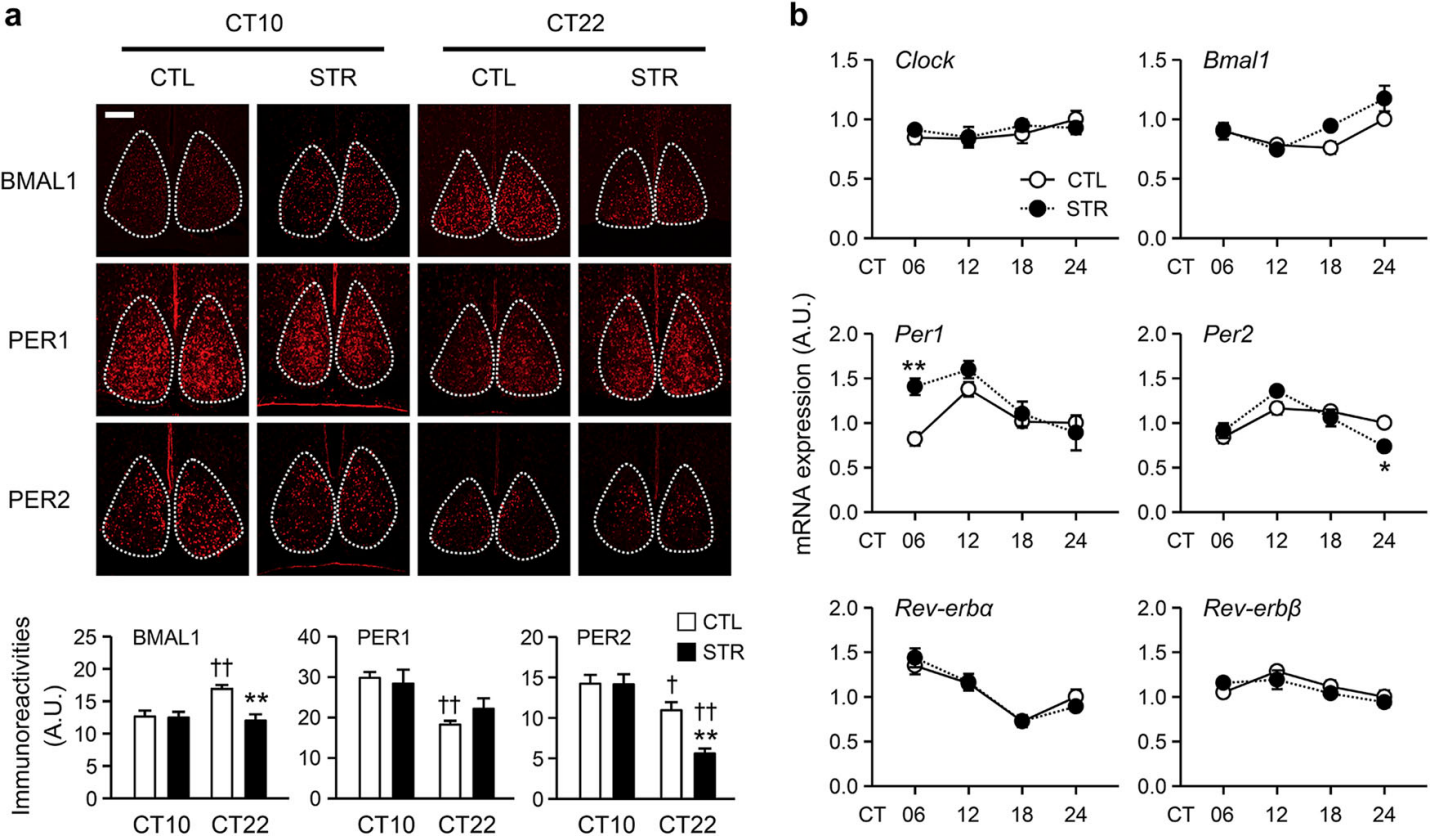
Expression levels of core clock genes in the SCN of adult male offspring. **a** Representative images of BMAL1, PER1, and PER2 protein expression in the SCN, which is marked by the dashed lines (scale bar = 50 µm). The fluorescence intensities of the indicated proteins in the SCN were normalized against the DAPI signal and are presented as the mean ± SE (*n* = 19–36 cells from three independent slices from different mice in each group; **p < 0.01 between CTL and STR at the corresponding time point; ^†^*p* < 0.05 and ^††^*p* < 0.01 between CT10 and CT22 by Bonferroni post hoc comparison). **b** mRNA expression profiles of clock genes in SCN-AH fragments from CTL and STR mice. The data were normalized against TATA-binding protein (TBP) expression levels and are expressed as the mean ± SE in arbitrary units (A.U.), with the mean CTL value at CT24 being defined as 1 (*n* = 4 for each time point; *p < 0.05 and **p < 0.01 between CTL and STR by Bonferroni post hoc test).

To examine whether the abnormal circadian molecular clock functions that were observed in the STR SCN explants were maintained in adult mice in vivo, we examined the TOD-related expression profiles of core clock components. IHC examination of the SCN demonstrated significant differences in BMAL1-, PER1-, and PER2-ir between circadian time (CT) 10 and CT22 (Fig. 3a; *p* < 0.05 for BMAL1 and *p* < 0.01 for PER1 and PER2 for the effect of TOD by two-way ANOVA; see Table S1 for the full ANOVA results). BMAL1- and PER2-ir were significantly different between CTL and STR mice (*p* < 0.01 for the effect of group on both BMAL1- and PER2-ir by two-way ANOVA; Table S1), and significant TOD × group interactions were found for these two clock proteins (*p* < 0.01 for BMAL1-ir and *p* < 0.05 for PER2-ir for the interaction effect by two-way ANOVA; Table S1). Bonferroni post hoc comparisons revealed significant alterations in BMAL1- and PER2-ir in the STR SCN at CT22. We then compared the mRNA expression profiles of *Clock, Bmal1, Per1, Per2, Rev-erbα*, and *Rev-erbβ* in the anterior hypothalamic fragments that contained the SCN (SCN-AH) between the STR and CTL mice at four different TODs (Fig. 3b). All examined clock genes except *Clock* exhibited significant TOD-dependent variations in their mRNA expression levels (Fig. 3b; *p* < 0.01 for *Bmal1, Per1, Per2, Rev-erbα* and *Rev-erbβ* for the effect of TOD by two-way ANOVA; see Table S2 for the full ANOVA results). Among them, the *Bmal1* and *Per1* showed significantly different expression profiles between groups (*p* < 0.05 for both *Bmal1* and *Per1* by two-way ANOVA; Table S2); however, only *Per1* and *Per2* mRNA levels were moderately altered in the STR SCN-AH compared with the CTL SCN-AH based on TOD-matched post hoc comparison (*p* < 0.01 for *Per1* at CT06; *p* < 0.05 for *Per2* at CT24).

### Behavioral rhythms of adult male STR mice

**Fig. 4:**
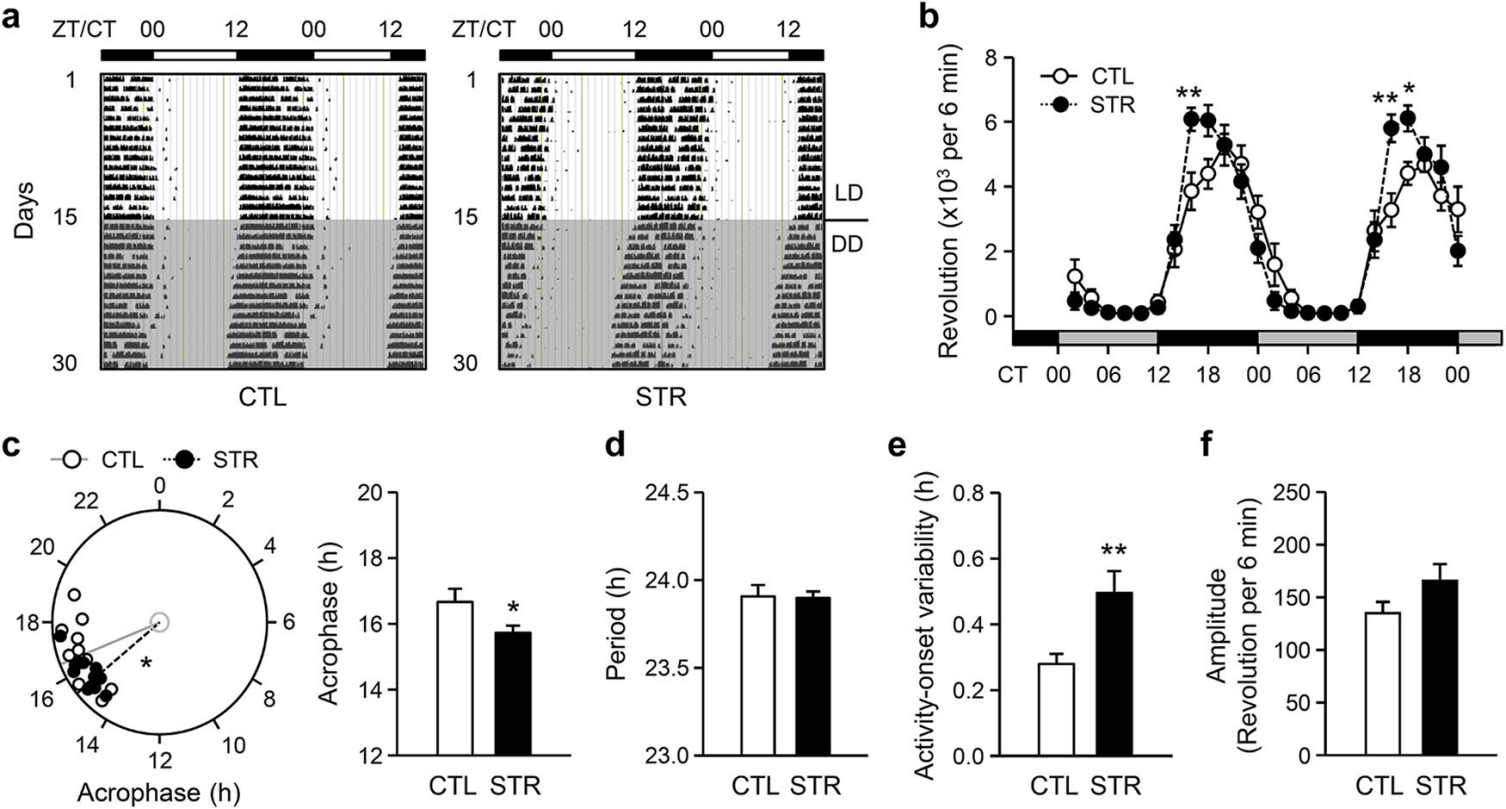
Altered behavioral rhythms in maternally stressed adult male mice. **a** Representative double-plotted actograms of the wheel-running activity of CTL and STR mice. **b** Mean wheel-running activity on the 4th and 5th days of DD conditions at 2-h intervals (*n* = 12 for each group; *p < 0.05 and **p < 0.01 between groups by Bonferroni post hoc comparison). **c** Phase angles of the wheel-running rhythm of each mouse (left) and the acrophase (right) are expressed as the mean ± SE for CTL and STR mice. **d**–**f** Period length (**d**), activity onset variability (**e**), and amplitude (**f**) of the circadian wheel-running activity of CTL and STR mice under DD conditions for 10 days from the 4th day after lights were turned off. The data in the bar charts are presented as the mean ± SE in the indicated units (*n* = 12 for each group; *p < 0.05 and **p < 0^.^01 between groups by Student’s *t*-test).

Despite the circadian abnormalities observed in the SCN cultures from STR mice, the clock gene expression profiles in the SCN of adult STR mice showed only modest alterations compared with those in the SCN of CTL mice. Therefore, we examined behavioral rhythms based on wheel-running activity, which is regarded as a primary functional output of the SCN central clock. Representative actograms are shown in Fig. 4a, and quantitative analyses on the 4th and 5th days of DD demonstrated a robust circadian rhythm of wheel-running activity in both CTL and STR mice (Fig. 4b; *F*_(23,506)_ = 69.94, *p* < 0.01 for the effect of TOD; *F*_(1,506)_ = 0.39, *p* = 0.54 for the effect of group; *F*_(23,506)_ = 3.94, *p* < 0.01 for the TOD × group interaction by two-way RM-ANOVA). Notably, Bonferroni post hoc comparison revealed significant group differences during the early phase of the subjective night, implying alterations in the phase and/or amplitude of behavioral rhythms in STR mice compared with CTL mice. Accompanying analysis of free-running rhythm in the DD period clearly demonstrated that the phase of the behavioral rhythm was significantly advanced in STR mice by approximately 1 h compared with that in CTL mice (Fig. 4c; 16.68 ± 0.39 h for CTL mice vs. 15.74 ± 0.21 h for STR mice, *p* < 0.05 by Student’s *t*-test). Furthermore, despite the lack of observed significant group differences for the free-running period (Fig. 4d; 23.91 ± 0.06 h for CTL mice vs. 23.90 ± 0.03 h for STR mice, *p* = 0.91), the activity onset variability was significantly increased in the STR mice compared with the CTL mice, in agreement with the altered SCN rhythm observed in the explant cultures prepared from 5-week-old mice (Fig. 4e; 0.28 ± 0.03 h for CTL mice vs. 0.50 ± 0.07 h for STR mice, *p* < 0.01). Although the amplitude of the activity of the STR mice tended to be higher and although there was an increase in wheel-running activity during the early nighttime, the difference between groups was not significant (Fig. 4f; 135.21 ± 10.73 revolutions per 6 min for CTL mice vs. 166.02 ± 15.80 revolutions per 6 min for STR mice, *p* = 0.12). These results suggest that the deleterious effects of prenatal stress, especially its effects on the phase and stability of central rhythms, may persist during postnatal development, even in animals reared under normal photoperiod conditions.

### Effects of maternal stress on adrenal and hepatic rhythms

**Fig. 5:**
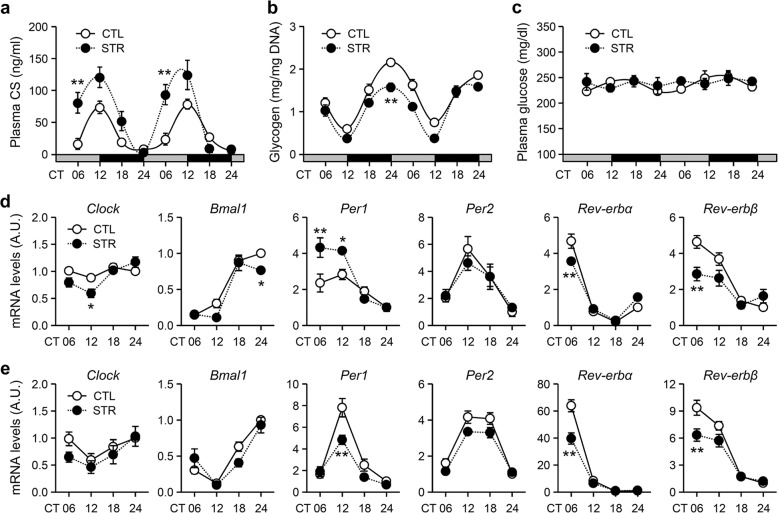
Influences of maternal stress on peripheral rhythms in adult male offspring. **a** Plasma CS concentration (*n* = 8 CTL mice and *n* = 6 STR mice at each point), **b** hepatic glycogen content (*n* = 7 CTL mice and *n* = 5 STR mice), and **c** plasma glucose levels (*n* = 8 CTL mice and *n* = 6 STR mice). The mice were housed under DD conditions and sacrificed at the indicated time points over a 2-d period. The data are presented as the mean ± SE in the indicated units (**p < 0.01 vs. CTL mice at the same time point by Bonferroni post hoc comparison). **d**, **e** mRNA expression profiles of clock genes in the adrenal gland (**d**) and liver (**e**). The data were normalized against TBP expression levels and are expressed as the mean ± SE in A.U., with the mean CTL value at CT24 being defined as 1 (*n* = 4 for each point, *p < 0.05 and **p < 0.01 vs. CTL mice at the same time point by Bonferroni post hoc correction).

The next set of experiments aimed to examine the effects of prenatal stress on peripheral circadian clocks. We primarily focused on the adrenal gland and liver, as the local clocks and rhythmic outputs of these tissues have been well characterized^13,14,26^. First, we compared the daily rhythm in plasma CS levels under DD conditions between adult male CTL and STR mice for two successive days starting on the 4th day. Similar to the behavioral rhythm results, compared with that of CTL mice, the circulating CS rhythm of STR mice exhibited an apparent phase advance and a significantly higher peak level around the activity onset time (Fig. 5a; *F*_(7,96)_ = 27.92, *p* < 0.01 for the effect of TOD; *F*_(1,96)_ = 35.64, *p* < 0.01 for the effect of group; *F*_(7,96)_ = 5.35, *p* < 0.01 for the interaction effect). The circadian glycogen content in liver tissues is negatively correlated with circulating CS levels throughout the day^27^. Indeed, in contrast with CS levels, hepatic glycogen content was associated with a reduced circadian amplitude (Fig. 5b; *F*_(7,80)_ = 52.04, *p* < 0.01 for the effect of TOD; *F*_(1,80)_ = 38.11, *p* < 0.01 for the effect of group; *F*_(7,80)_ = 2.00, *p* = 0.06 for the interaction effect), although the plasma glucose levels under free-feeding conditions were not significantly different between groups (Fig. 5c; *F*_(7,96)_ = 1.00, *p* = 0.43 for the effect of TOD; *F*_(1,96)_ = 0.50, *p* = 0.48 for the effect of group; *F*_(7,96)_ = 0.75, *p* = 0.63 for the interaction effect). In addition to endocrine and metabolic parameters, we also examined the mRNA expression profiles of canonical clock genes in the adrenal gland (Fig. 5d) and liver (Fig. 5e). All tested clock genes exhibited significant TOD-dependent alterations in both tissues (*p* < 0.01 for the effect of TOD by two-way ANOVA; see Tables S3 and S4 for the full ANOVA results). There were significant group differences in the mRNA expression levels of *Clock, Bmal1, Per1*, and *Rev-erbβ* in the adrenal gland (*p* < 0.05 for *Clock* and *Rev-erbβ*; *p* < 0.01 for *Bmal1* and *Per1*; Table S3) and of *Per1, Per2, Rev-erbα*, and *Rev-erbβ* in the liver (*p* < 0.01 for the effect of group by two-way ANOVA; Table S4). Notably, compared with those in the adrenal glands of CTL mice, *Per1* mRNA expression levels in the adrenal glands of STR mice were increased during subjective daytime, whereas these levels were significantly lower in the liver at CT12, as evaluated by post hoc comparisons. *Rev-erbα* and *Rev-erbβ* mRNA expression levels were significantly reduced in both tissues at CT06 in STR mice compared with CTL mice. These results suggest that maternal stress may also affect the rhythmic functions of local clocks in peripheral tissues, such as the adrenal gland and liver, through either SCN-dependent or SCN-independent mechanisms.

## Discussion

The present study clearly demonstrated that maternal stress attenuated the amplitude of cyclic mPer1::LUC expression in cultured SCN explants via a mechanism that was associated with impaired synchrony among individual SCN cells, implying that intrinsic defects in the master oscillator were caused by prenatal exposure to stress. Furthermore, maternal stress had a long-lasting influence on the behavioral and physiological rhythms of offspring as well as on the TOD-dependent clock gene expression profiles in the SCN, adrenal gland, and liver. Indeed, compared with adult CTL mice, adult STR mice exhibited impaired period stability, significant phase enhancements of their behavioral rhythms, and a more robust CS rhythm in circulation with abnormally enhanced secretion that coincided with an advanced activity onset time. These findings collectively support the idea that the circadian system, including its central pacemaker, is a key target of the programming effects caused by exposure to stress in the womb.

The potential for perinatal crosstalk between circadian and stress systems has been recognized in rodent models as well as in human epidemiological studies^28,29^, but the underlying mechanisms remain elusive. For example, earlier studies reported that stress in utero advances the diurnal phases of CS profiles and locomotor activity in adult rats under a normal LD photoperiod^18,19^. We employed ex vivo SCN explant cultures to examine whether maternal stress has an impact on the intrinsic properties of the central oscillator. As shown in Fig. 1, compared with those from CTL mice, whole SCN explants from neonatal STR mPer1::LUC transgenic mice exhibited a shortened period and lower amplitude of bioluminescence signals. These results support the idea that maternal stress causes a developmental defect in the central clocks of pups. The impaired amplitude of cyclic mPer1::LUC expression persisted in STR SCN cultures prepared from 5-week-old mice, although the difference in period was abolished during postnatal development. In mice, neurogenesis in the developing SCN primarily occurs between embryonic days 10 and 15 (E10 and 15), and intra-SCN circuits are formed during the following days^30^. Retinal projections then reach the SCN shortly after birth, and functional photoreception begins at approximately postnatal week 1^31^. Therefore, our maternal stress scheme, which began at 8.5 dpc, may have influenced both neurogenesis and intra-SCN circuit formation. The reduced amplitude of circadian mPer1::LUC activity in the STR SCN may arise from multiple causes, such as the epigenetic programming of core clock genes, including *Per1*; a reduced number of pacemaker neurons; or desynchrony among SCN cells. However, the first two possibilities can be excluded because the amplitude of bioluminescence signals in individual SCN cells was even higher in neonatal STR cultures, and there were no apparent defects in the gross anatomy of the stressed SCN. Instead, STR SCN cultures showed significantly larger variations among individual cells than CTL SCN cultures. Intra-SCN circuits and intercellular communication among SCN cells provide the neuroanatomical and neurochemical bases of the robustness of the SCN central oscillator^32^. The cellular synchrony of clock gene transcription across numerous pacemaker neurons is supported by the coupling of neural activities among individual cells in the SCN^33^. Mechanical dissociation also supports this notion. For example, dissociated SCN neurons from *Per1*-deficient or *Cry1*-deficient mice lose cell-autonomous mPer2::LUC rhythm, whereas whole SCN explants from the same mutant mice maintain this rhythm^34^. Therefore, maternal stress most likely exerts deleterious effects on the developing SCN by disturbing intra-SCN circuit formation required for synchrony among pacemaker cells rather than impairing the molecular clockwork of individual SCN cells.

Nonetheless, these intrinsic defects cannot fully account for the circadian dysfunctions observed in the adult offspring. In accordance with the SCN culture results, circadian instability is evident in the behavioral rhythms of STR offspring, which are tightly linked with the circadian activities of the SCN^11,35^. Furthermore, circadian wheel-running activity as well as CS secretion from the adrenal gland, which is also under the relatively tight control of the SCN compared with other peripheral rhythms^13,15,36^, exhibited significant phase advances. However, the locomotor activity of the STR mice showed a normal amplitude, even under DD conditions, suggesting the presence of a certain postnatal compensatory mechanism. Although neural properties and the molecular clock machinery are prenatally established, the SCN undergoes postnatal maturation, particularly when forming connections with retinal projections and photoreception to produce physiological and behavioral rhythms in response to systemic zeitgebers^31,37^. It is thus possible that the impaired synchrony of SCN pacemaker neurons could be rescued during postnatal maturation in concert with the formation of neural connections with extra-SCN regions. On the other hand, it is also plausible that the abnormal circadian traits in the STR SCN, as observed at the level of mPer1::LUC activity, may not be sufficient to evoke consistent circadian dysfunction at the systemic level. Nevertheless, the mRNA and protein expression levels of core clock genes in the hypothalamic SCN were significantly altered in adult STR mice compared with adult CTL mice, as evidenced by the increase in *Per1* mRNA expression during subjective daytime. These results provide additional support for the idea that maternal stress has long-term consequences on SCN functions in adult offspring to some extent.

Hyperactivity of the HPA axis associated with reduced sensitivity to the negative feedback actions of GCs in adult offspring is a well-known consequence of prenatal stress^3,4^. Our results showed that maternal stress, in addition to programming stress reactivity, can program the hypersecretion of adrenal CS in a TOD-dependent manner, particularly during the rising phase of circadian CS regulation. In accordance, the area under the curve of the circadian CS profiles appeared to be much larger in STR mice than in CTL mice (Fig. 5a). The mRNA expression levels of negative GC-responsive element (nGRE)-containing clock genes, such as *Clock* and *Rev-erbα*^38,39^, were reduced in the adrenal gland and liver but not in the SCN-AH, which is less responsive to GCs in circulation due to lower expression of canonical GC receptors^16,17^. Notably, circadian changes in hepatic glycogen content were significantly attenuated in STR animals, and this result was in contrast with the CS rhythm. The stress-induced secretion of or pathological increase in GCs is known to increase the storage of glycogen in the liver and to increase gluconeogenesis^40^; however, plasma GC levels and hepatic glycogen content follow comparatively antiphasic circadian oscillations in rodents^27^, suggesting that a more complicated mechanism exists for the circadian regulation of glycogen homeostasis in the liver. Interestingly, similar hepatic glycogen and plasma glucose profiles as those observed in our STR mice under free-feeding conditions have been reported in clock-defective ClockΔ19 mutant mice housed under a normal LD photoperiod^41^. Considering that all tested clock genes under the direct control of E-box-mediated transcription showed decreased mRNA expression that coincided with the peak time in the STR liver, hormonal and clock-dependent regulation may cooperate to control hepatic glycogen levels in a circadian manner. These findings suggest that circadian CS hypersecretion, in addition to the well-known hyperactivity in response to stress, may contribute to the GC-mediated deleterious physiological and behavioral consequences of maternal stress that were observed in offspring in association with programming effects on the circadian clock system.

Furthermore, it is reasonable to surmise that the hyperactivity of adrenal CS secretion in STR mice may have a certain impact on the impaired rhythmicity of the SCN as well as on circadian physiology and behavior. Considering the widespread roles of GCs in the harmonization of peripheral rhythms^10,14^, some of the circadian traits, of STR offspring, such as metabolic phenotypes, can be directly affected by the alteration of CS rhythm. It is also plausible that the phase of locomotor rhythm can be influenced by advanced GC profiles, as CS rhythm has been shown to regulate the speed of behavioral re-entrainment under conditions of experimental jet lag^17^. However, it remains unclear whether the altered GC rhythm underlies the impaired rhythmicity of the stressed SCN as well as unstable behavioral rhythms. First, the SCN has been presumed to be less sensitive to GC signaling due to the lower expression of GC receptors, as mentioned above. For example, repeated injection of an exogenous GC for 7 days around the activity onset time does not elicit significant effects on circadian locomotor activity or body temperature^42^. Furthermore, additional CS peaks induced by a restricted daytime feeding regimen for 7–10 days barely affect clock gene expression in the SCN and yield only a small amount of SCN-independent food-anticipatory activity compared with SCN-driven locomotor activity during the active period, whereas this feeding regimen leads to drastic phase shifts of cyclic gene expression in numerous peripheral tissues^15,43^. It is rather remarkable that circadian instabilities in both cyclic mPer1::LUC expression in the cultured SCN and locomotor activity of the STR mice are observed. The unstable SCN rhythm observed ex vivo remained obvious in cultures prepared from 5-week-old offspring, corresponding to the age at which the HPA axis becomes sufficiently mature^1,3^. These findings suggest that the maternally stressed SCN harbors intrinsic defects that are independent of the hormonal milieu and that this instability of the central rhythm could be manifested as impaired stability of overt rhythms.

In conclusion, we provided direct evidence that support the notion that maternal stress during gestation has profound effects on the developing SCN and has long-term consequences on the circadian system of offspring. Both clinical and animal studies have suggested a close relationship between prenatal stress and the development of diseases later in life^1,2^. For example, growth retardation caused by maternal stress or placental insufficiency has been associated with a higher risk of cardiometabolic diseases, such as hypertension, obesity, and diabetes mellitus. Prenatal stress has also been linked with an increased incidence of cognitive impairment, depressive disorders, and schizophrenia. Importantly, sleep and circadian disruptions are widely observed in association with these diseases^44,45^. Furthermore, growing evidence points to a causal role for circadian dysfunctions in the onset and symptoms of circadian rhythm-related metabolic^26,46,47^ and neuropsychiatric diseases^48–50^. Therefore, dysfunction of the SCN central clock and misregulation of the circadian timing system may play roles in mediating the various adverse effects of maternal stress on the physiology and mental health of offspring. In this context, behavioral or pharmacological interventions to correct circadian disruptions could be applicable for ameliorating the pathological conditions programmed by stress experienced in the womb.

## Supplementary information

Supplementary tables
