# Supplementary material for: Programming effects of maternal stress on the circadian system of adult offspring
Source: Exp Mol Med. 2020 Mar 11;52(3):473–84. doi: 10.1038/s12276-020-0398-9 (PMC7156466; doi:10.1038/s12276-020-0398-9)
Supplement: Supplementary file 1 — Supplementary tables [file 12276_2020_398_MOESM1_ESM.docx]

**Table S1. Full results of two-way ANOVA related to Figure 3a**

| **Targets** | ***F*- and *P*-values by ANOVA** | ***P*-value by Bonferroni comparison** |
| --- | --- | --- |
| **BMAL1-ir** | F_(1, 126)_ = 5.110, ^*^p < 0.05 for TOD  F_(1, 126)_ = 9.121, ^**^p < 0.01 for group  F_(1, 126)_ = 7.930, ^**^p < 0.01 for interaction | p > 0.05 for CTL *vs.* STR at CT10  ^**^p < 0.01 for CTL *vs.* STR at CT22  ^**^p < 0.01 for CT10 *vs.* CT22 in CTL  p > 0.05 for CT10 *vs.* CT22 in STR |
| **PER1-ir** | F_(1, 94)_ = 19.66, ^**^p < 0.01 for TOD  F_(1, 94)_ = 0.3783, p > 0.05 for group  F_(1, 94)_ = 1.776, p > 0.05 for interaction | p > 0.05 for CTL *vs.* STR at CT10  p > 0.05 for CTL *vs.* STR at CT22  ^**^p < 0.01 for CT10 *vs.* CT22 in CTL  p > 0.05 for CT10 *vs.* CT22 in STR |
| **PER2-ir** | F_(1, 114)_ = 34.46, ^**^p < 0.01 for TOD  F_(1, 114)_ = 7.296, ^**^p < 0.01 for group  F_(1, 114)_ = 6.866, ^*^p < 0.05 for interaction | p > 0.05 for CTL *vs.* STR at CT10  ^**^p < 0.01 for CTL *vs.* STR at CT22  ^*^p < 0.05 for CT10 *vs.* CT22 in CTL  ^**^p < 0.01 for CT10 *vs.* CT22 in STR |

**Table S2. Full results of two-way ANOVA related to Figure 3b**

| **Targets** | ***F*- and *P*-values by ANOVA** | ***P*-value by Bonferroni comparison^a^** |
| --- | --- | --- |
| ***Clock*** | F_(3, 24)_ = 1.400, p = 0.2670 for TOD  F_(1, 24)_ = 0.1876, p = 0.6688 for group  F_(3, 24)_ = 0.6012, p = 0.6205 for interaction | p > 0.05 for CTL *vs.* STR at CT06  p > 0.05 for CTL *vs.* STR at CT12  p > 0.05 for CTL *vs.* STR at CT18  p > 0.05 for CTL *vs.* STR at CT24 |
| ***Bmal1*** | F_(3, 24)_ = 12.28, ^**^p < 0.01 for TOD  F_(1, 24)_ = 4.438, ^*^p < 0.05 for group  F_(3, 24)_ = 2.122, p = 0.1238 for interaction | p > 0.05 for CTL *vs.* STR at CT06  p > 0.05 for CTL *vs.* STR at CT12  p > 0.05 for CTL *vs.* STR at CT18  p > 0.05 for CTL *vs.* STR at CT24 |
| ***Per1*** | F_(3, 24)_ = 9.083, ^**^p < 0.01 for TOD  F_(1, 24)_ = 6.316, ^*^p < 0.05 for group  F_(3, 24)_ = 3.559, ^*^p < 0.05 for interaction | ^**^p < 0.01 for CTL *vs.* STR at CT06  p > 0.05 for CTL *vs.* STR at CT12  p > 0.05 for CTL *vs.* STR at CT18  p > 0.05 for CTL *vs.* STR at CT24 |
| ***Per2*** | F_(3, 24)_ = 15.63, ^**^p < 0.01 for TOD  F_(1, 24)_ = 0.1325, p = 0.7191 for group  F_(3, 24)_ = 4.299, ^*^p < 0.05 for interaction | p > 0.05 for CTL *vs.* STR at CT06  p > 0.05 for CTL *vs.* STR at CT12  p > 0.05 for CTL *vs.* STR at CT18  ^*^p < 0.05 for CTL *vs.* STR at CT24 |
| ***Rev-erbα*** | F_(3, 24)_ = 28.78, ^**^p < 0.01 for TOD  F_(1, 24)_ = 0.0007, p = 0.9797 for group  F_(3, 24)_ = 0.5776, p = 0.6353 for interaction | p > 0.05 for CTL *vs.* STR at CT06  p > 0.05 for CTL *vs.* STR at CT12  p > 0.05 for CTL *vs.* STR at CT18  p > 0.05 for CTL *vs.* STR at CT24 |
| ***Rev-erbβ*** | F_(3, 24)_ = 6.003, ^**^p < 0.01 for TOD  F_(1, 24)_ = 0.4740, p = 0.4977 for group  F_(3, 24)_ = 1.072, p = 0.3796 for interaction | p > 0.05 for CTL *vs.* STR at CT06  p > 0.05 for CTL *vs.* STR at CT12  p > 0.05 for CTL *vs.* STR at CT18  p > 0.05 for CTL *vs.* STR at CT24 |

^a^*P*-values between CTL and STR at the same time-of-day were presented here.

**Table S3. Full results of two-way ANOVA related to Figure 5d**

| **Targets** | ***F*- and *P*-values by ANOVA** | ***P*-value by Bonferroni comparison^a^** |
| --- | --- | --- |
| ***Clock*** | F_(3, 24)_ = 12.79, ^**^p < 0.01 for TOD  F_(1, 24)_ = 4.793, ^*^p < 0.05 for group  F_(3, 24)_ = 5.223, ^**^p < 0.01 for interaction | p > 0.05 for CTL *vs.* STR at CT06  ^*^p < 0.05 for CTL *vs.* STR at CT12  p > 0.05 for CTL *vs.* STR at CT18  p > 0.05 for CTL *vs.* STR at CT24 |
| ***Bmal1*** | F_(3, 24)_ = 119.5, ^**^p < 0.01 for TOD  F_(1, 24)_ = 9.177, ^**^p < 0.01 for group  F_(3, 24)_ = 2.659, p = 0.0711 for interaction | p > 0.05 for CTL *vs.* STR at CT06  p > 0.05 for CTL *vs.* STR at CT12  p > 0.05 for CTL *vs.* STR at CT18  ^*^p < 0.05 for CTL *vs.* STR at CT24 |
| ***Per1*** | F_(3, 24)_ = 30.80, ^**^p < 0.01 for TOD  F_(1, 24)_ = 10.34, ^**^p < 0.01 for group  F_(3, 24)_ = 6.331, ^**^p < 0.01 for interaction | ^**^p < 0.01 for CTL *vs.* STR at CT06  ^*^p < 0.05 for CTL *vs.* STR at CT12  p > 0.05 for CTL *vs.* STR at CT18  p > 0.05 for CTL *vs.* STR at CT24 |
| ***Per2*** | F_(3, 24)_ = 16.58, ^**^p < 0.01 for TOD  F_(1, 24)_ = 0.1123, p = 0.7405 for group  F_(3, 24)_ = 0.5158, p = 0.6754 for interaction | p > 0.05 for CTL *vs.* STR at CT06  p > 0.05 for CTL *vs.* STR at CT12  p > 0.05 for CTL *vs.* STR at CT18  p > 0.05 for CTL *vs.* STR at CT24 |
| ***Rev-erbα*** | F_(3, 24)_ = 217.4, ^**^p < 0.01 for TOD  F_(1, 24)_ = 0.5396, p = 0.4697 for group  F_(3, 24)_ = 9.620, ^**^p < 0.01 for interaction | ^**^p < 0.01 for CTL *vs.* STR at CT06  p > 0.05 for CTL *vs.* STR at CT12  p > 0.05 for CTL *vs.* STR at CT18  p > 0.05 for CTL *vs.* STR at CT24 |
| ***Rev-erbβ*** | F_(3, 24)_ = 32.10, ^**^p < 0.01 for TOD  F_(1, 24)_ = 7.515, ^*^p < 0.05 for group  F_(3, 24)_ = 5.342, ^**^p < 0.01 for interaction | ^**^p < 0.01 for CTL *vs.* STR at CT06  p > 0.05 for CTL *vs.* STR at CT12  p > 0.05 for CTL *vs.* STR at CT18  p > 0.05 for CTL *vs.* STR at CT24 |

^a^*P*-values between CTL and STR at the same time-of-day were presented here.

**Table S4. Full results of two-way ANOVA related to Figure 5e**

| **Targets** | ***F*- and *P*-values by ANOVA** | ***P*-value by Bonferroni comparison^a^** |
| --- | --- | --- |
| ***Clock*** | F_(3, 24)_ = 4.796, ^**^p < 0.01 for TOD  F_(1, 24)_ = 2.522, p = 0.1253 for group  F_(3, 24)_ = 0.6789, p = 0.5735 for interaction | p > 0.05 for CTL *vs.* STR at CT06  p > 0.05 for CTL *vs.* STR at CT12  p > 0.05 for CTL *vs.* STR at CT18  p > 0.05 for CTL *vs.* STR at CT24 |
| ***Bmal1*** | F_(3, 24)_ = 48.00, ^**^p < 0.01 for TOD  F_(1, 24)_ = 0.5426, p = 0.4685 for group  F_(3, 24)_ = 2.478, p = 0.0855 for interaction | p > 0.05 for CTL *vs.* STR at CT06  p > 0.05 for CTL *vs.* STR at CT12  p > 0.05 for CTL *vs.* STR at CT18  p > 0.05 for CTL *vs.* STR at CT24 |
| ***Per1*** | F_(3, 24)_ = 58.49, ^**^p < 0.01 for TOD  F_(1, 24)_ = 10.66, ^**^p < 0.01 for group  F_(3, 24)_ = 4.787, ^**^p < 0.01 for interaction | p > 0.05 for CTL *vs.* STR at CT06  ^**^p < 0.01 for CTL *vs.* STR at CT12  p > 0.05 for CTL *vs.* STR at CT18  p > 0.05 for CTL *vs.* STR at CT24 |
| ***Per2*** | F_(3, 24)_ = 83.98, ^**^p < 0.01 for TOD  F_(1, 24)_ = 9.419, ^**^p < 0.01 for group  F_(3, 24)_ = 1.773, p = 0.1791 for interaction | p > 0.05 for CTL *vs.* STR at CT06  p > 0.05 for CTL *vs.* STR at CT12  p > 0.05 for CTL *vs.* STR at CT18  p > 0.05 for CTL *vs.* STR at CT24 |
| ***Rev-erbα*** | F_(3, 24)_ = 253.2, ^**^p < 0.01 for TOD  F_(1, 24)_ = 17.38, ^**^p < 0.01 for group  F_(3, 24)_ = 15.02, ^**^p < 0.01 for interaction | ^**^p < 0.01 for CTL *vs.* STR at CT06  p > 0.05 for CTL *vs.* STR at CT12  p > 0.05 for CTL *vs.* STR at CT18  p > 0.05 for CTL *vs.* STR at CT24 |
| ***Rev-erbβ*** | F_(3, 24)_ = 88.59, ^**^p < 0.01 for TOD  F_(1, 24)_ = 9.762, ^**^p < 0.01 for group  F_(3, 24)_ = 4.409, ^*^p < 0.05 for interaction | ^**^p < 0.01 for CTL *vs.* STR at CT06  p > 0.05 for CTL *vs.* STR at CT12  p > 0.05 for CTL *vs.* STR at CT18  p > 0.05 for CTL *vs.* STR at CT24 |

^a^*P*-values between CTL and STR at the same time-of-day were presented here.
